# Isolation and characterization of the mitochondrial genome of *Gymnodraco acuticeps* (Perciformes: Bathydraconidae) with phylogenetic consideration

**DOI:** 10.1080/23802359.2017.1361361

**Published:** 2017-08-16

**Authors:** Wei Song, Lingzhi Li, Hongliang Huang, Fenfang Chen, Ming Zhao, Keji Jiang, Fengying Zhang, Chunyan Ma, Xuezhong Chen, Lingbo Ma

**Affiliations:** a Key Laboratory of Oceanic and Polar Fisheries, Ministry of Agriculture, East China Sea Fisheries Research Institute, Chinese Academy of Fishery Sciences, Shanghai, China;; b College of Fisheries and Life Sciences, Shanghai Ocean University, Shanghai, China

**Keywords:** *Gymnodraco acuticeps*, mito-genome, genome structure, phylogenetic

## Abstract

*Gymnodraco acuticeps*is is an Antarctic fish living in the Southern Ocean. Until now, studies on *G. acuticeps* are still limited. As an Antarctic fish, obtaining and characterization of the mitochondrial genome of *G. acuticeps* will be important for elucidation of the mechanism of cold-adapting evolution in mitochondrion. In this study, we first isolated and characterized the mitochondrial genome sequence of *G. acuticeps* with 15,987 bp in length. It contained of 34 genes (12 protein-coding genes, 20 transfer RNA genes, 2 ribosomal RNA genes) and a partial putative control region. Gene organization and nucleotide composition of obtained mito-genome were similar to those of other Antarctic fish. Twenty-eight genes were encoded by heavy strand, while six genes were encoded by light strand. Further, the phylogenetic tree, which based on 12 protein-coding genes, revealed that the *G. acuticeps* was genetically closest to species *Parachaenichthys charcoti* among 18 species. We hope this work would be helpful for the population genetics and molecular evolution studies.

The family Bathydraconidae of the suborder Notothenioidei was consisted of at least 11 genera and 16 species living in the south of the Antarctic Polar Front. *Gymnodraco acuticeps* belongs to family Bathydraconidae which lived beneath the sea ice of the Southern Ocean (Eastman and Hikida 1991). It experience unusual environmental conditions, including highly oxygenated subzero water. The common name of *G. acuticeps* is the naked dragonfish, which stems from its body feature such as lack of scales, the dragon-like form of its head, a protruding lower jaw bearing prominent, and the exposed canines. They are the predators in Antarctic food web, feeding on a variety of organisms including some fish, amphipods and polychaetes (Evans et al. 2005). Some regions of the mitochondrial genome were thought to be the ideal markers for studies on population genetic diversity, molecular phylogeny, and species identification due to the high mutation rate, simple structure, abundant distribution and maternal inheritance. So far, mitochondrial genome sequence of *G. acuticeps* is still unavailable and this has hampered the genetic study in this group fishes.

Adult fish of *G. acuticeps* was collected near Zhongshan Station (68tio 708tio after freezing at −80 °C), it was transported to East China Sea Fisheries Research Institue, Chinese Academy of Fishery Science for storage and DNA extraction. In this study, we determined the nearly complete mitochondrial genome of *G. acuticeps* (the accession number: KX840362). This genome was 15,987 bp in length, including 12 protein-coding genes, 20 tRNA genes, two rRNA genes. A non-coding region with high A + T content between tRNA^Pro^ and tRNA^Phe^ was identified as the putative control region. Recently, the mitochondrial gene such as ND6 and tRNA^Glu^ were found to be ‘lost’ in Antarctic notothenioids (Papetti et al. 2007). Later they were found between duplicated control regions (CRs) in rearranged mitochondrial genomes of this taxon (Zhuang and Cheng 2010). The structure and gene arrangement of obtained mitochondrial genome of *G. acuticeps* were similar with those obtained from other fish species such as *Chionodraco hamatus* (Song et al. 2016), *Notothenia coriiceps* and *Notothenia rossii* (Zhuang and Cheng 2010). The total nucleotide composition of G*. acutips* is mitogenome was 25.11% for A, 17.40% for G, 30.80% for C, and 26.69% for T, with a high A + T content of 51.80%. Twelve protein-coding genes were 10,948 bp in length and the composition was 23.44% for A, 16.72% for G, 31.91% for C and 27.93% for T, with an A + T content of 51.37%.

Among 34 genes, 28 were encoded by the heavy strand (H-strand) and only six genes (tRNA^Gln^, tRNA^Ala^, tRNA^Asn^, tRNA^Cys^, tRNA^Tyr^ and tRNA^Ser^) were encoded by light strand (L-strand). 11 protein-coding genes were started by ATG, while the other protein-coding gene COI was initiated by GTG. In total, four types of stop codons (TAG, TAA, TAG, and T-) were detected in these 12 protein-coding genes, and the gene ND5 was stopped by a rare stop codon TAG. A total of 14 intergenic spacers were found in this genome, 10 on H-strand, and four on L-strand with the ranges from 1 to 44 bp. Meanwhile, six overlaps were observed, with the lengths between 1 to 10 bp. The longest overlap (10 bp) occurred between ATP8 and ATP6, while the biggest intergenic spacer (44 bp) was located between tRNA^Asn^ and tRNA^Cys^. The putative origin of light strand replication was found between tRNA^Asn^ and tRNA^Cys^, with a length of 44 bp.

The phylogenetic relationship of *G. acuticeps* within 13 Antarctic fish was analyzed using 12 concatenated protein-coding genes except ND6 (Figure 1). Usually, ND6 was not used for phylogenetic analysis due to its high heterogeneity and poor phylogenetic performance (Miya and Nishida 2000). The phylogenetic tree was reconstructed using neighbor-joining (NJ) algorithms in MEGA 4.0 software with 1000 bootstrap replicates (Zardoya and Meyer, 1996). From the tree topologies, we can find that the *G. acuticeps* was genetically closest to species *Parachaenichthys charcoti* among 16 species. This result is identical to previous phylogeny studies by using the partial mitochondrial gene (Bargelloni et al. 1994).

**Figure 1.**
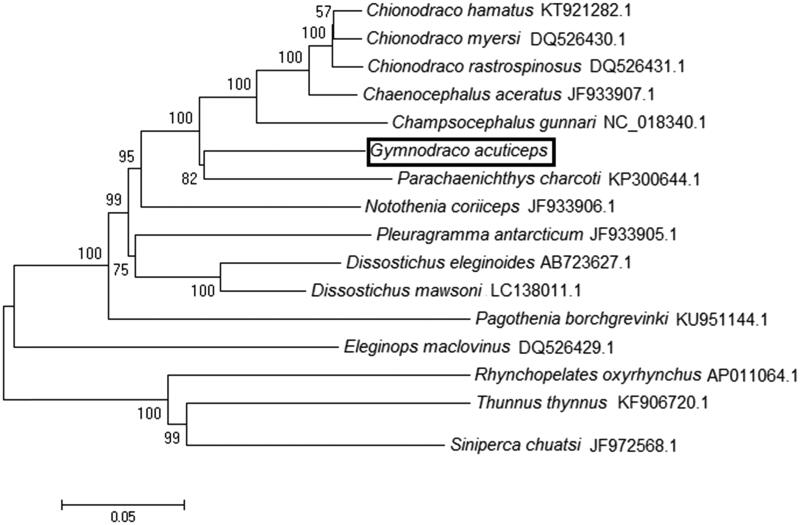
The phylogenetic relationship of *G. acuticeps* within Antarctic fish based on 12 protein-coding genes.
